# Advances in the Preparation, Stability, Metabolism, and Physiological Roles of Anthocyanins: A Review

**DOI:** 10.3390/foods12213969

**Published:** 2023-10-30

**Authors:** Qi Li, Fengzhen Zhang, Zhenzhen Wang, Yaoze Feng, Yahong Han

**Affiliations:** 1 Department of Neurosurgery, Union Hospital, Tongji Medical College, Huazhong University of Science and Technology, Wuhan 430022, China; 2 School of Public Health, Wuhan University, Wuhan 430071, China; 3 Key Laboratory of Aquaculture Facilities Engineering, Ministry of Agriculture and Rural Affairs, College of Engineering, Huazhong Agricultural University, Wuhan 430070, China; yaoze.feng@mail.hzau.edu.cn; 4 College of Food Science and Engineering, Wuhan Polytechnic University, Wuhan 430023, China

**Keywords:** anthocyanins, extraction, structure, anti-degradation, bioavailability, prevent disease

## Abstract

Anthocyanins are natural flavonoid polyphenolic compounds widely found in fruits and vegetables. They exhibit antioxidant properties and prophylactic effects in the immune and cardiovascular systems, confer protection against cancer, and contribute to the prevention of cardiovascular diseases. Thus, their incorporation into functional foods, pharmaceuticals, supplements, and cosmetic formulations aims at promoting human well-being. This review comprehensively outlined the structural attributes of anthocyanins, expanding upon diverse methodologies employed for their extraction and production. Additionally, the stability, metabolic pathways, and manifold physiological functions of anthocyanins were discussed. However, their constrained fat solubility, susceptibility to instability, and restricted bioavailability collectively curtail their applicability and therapeutic efficacy. Consequently, a multidimensional approach was imperative, necessitating the exploration of innovative pathways to surmount these limitations, thereby amplifying the utilitarian significance of anthocyanins and furnishing pivotal support for their continual advancement and broader application.

## 1. Introduction

Anthocyanins are a class of natural pigments renowned for their bright color and health-promoting attributes, conferring multifaceted benefits to human well-being [1]. These compounds showed robust antioxidant properties, orchestrating a modulation of oxidative stress and inflammatory responses [2]. Furthermore, their cancer-preventive potential, cardiovascular regulatory capacity, vision enhancement, as well as antibacterial and anti-inflammatory functionalities have been substantiated [3]. Given the diverse physiological functions of anthocyanins, they were widely applied to functional foods, supplements, and pharmaceuticals. Various analytical methodologies, encompassing ultra-high performance liquid chromatography (UHPLC) [4], UV-visible spectroscopy, and liquid chromatography [5], among others, have been used in unveiling the intricate structural and compositional facets of anthocyanins. The techniques used for anthocyanin extraction and synthesis are also diverse and important. Although the sources, extraction, and characterization were previously summarized [6], this review not only delved into these characterization and preparation avenues and scrutinized their merits and limitations, but also comprehensively discussed the stability, metabolism, and physiological function of anthocyanins.

The stability of anthocyanins is relatively weak, as their susceptibility to degradation under light and during thermal food processing [7]. To combat this, bolstering anthocyanin stability necessitated a comprehensive exploration of factors impacting their degradation, coupled with structural modifications to preserve both color integrity and nutritional potency. The low bioavailability of anthocyanins underscored the importance of comprehending their digestion, absorption, and metabolic dynamics within the human body, a facet that is dissected in subsequent sections [8].

**Figure 1:**
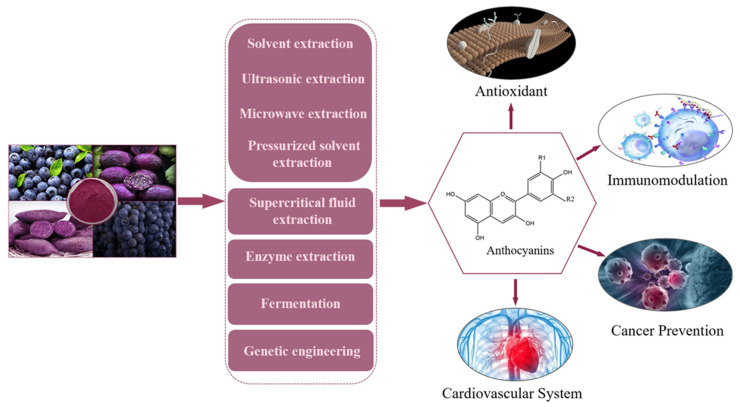
Schematic diagram of preparation and physiological function of anthocyanins.

This paper discussed varied preparation methodologies including solvent extraction, ultrasonic extraction, and genetic engineering, all of which endowed anthocyanins with pivotal physiological attributes, spanning antioxidative potential, immunomodulation, cancer mitigation, and cardiovascular homeostasis (Figure 1). This comprehensive study amalgamated insights into the structural attributes of anthocyanins, their preparation protocols, stability profiles, in vivo metabolic trajectories, and an array of physiological functionalities. By encapsulating these multifarious dimensions, this article aspired to lay a robust theoretical foundation to propel the application of anthocyanins across diverse domains, encompassing food, medical intervention, cosmetics, etc.

## 2. Molecular Structure of Anthocyanins

The molecular structure of anthocyanins varies, owing to their widespread presence in the cells of numerous plant species, spanning 27 families and 73 genera, and encompassing diverse plant organs such as roots, stems, leaves, and fruits [9]. Anthocyanins, extensively identified in sources like grape seeds, blueberries, purple kale, hawthorn, and tea leaves, exhibit a distinctive framework denoted as the C_6_-C_3_-C_6_ skeleton structure. Anthocyanins are a class of flavonoid polyphenol compounds characterized by a 2-phenylchromen ring adorned with various substituents [10].

The basic structure of the anthocyanins consists of two benzene rings, namely A and B, tethered together by an oxygenated pyrene ring, typically referred to as ring C. The 3-, 5-, and 7-carbon positions of the anthocyanin moiety often feature substituted hydroxyl groups. It is the assortment of substituents at the R_1_ and R_2_ carbon positions that gives rise to a plethora of anthocyanin varieties. Remarkably, over 20 distinct anthocyanin types have been identified, with particular focus on six extensively investigated ones: Pelargonidin (Pg), Cyanidin (Cy), Delphinidin (Dp), Peonidin (Pn), Petunidin (Pt), and Malvidin (My) [11].

Owing to their multiple phenolic hydroxyl groups, anthocyanins predominantly exist in a glycosidic form, commonly bonded to one or more sugars such as glucose, galactose, or xylose. These glycosidic bonds typically form at the C_3_ position of the C ring and the C_5_ and C_7_ positions of the A ring. Furthermore, beyond glycosylation, anthocyanins have the capacity to engage in acylation, a chemical process in which they bind with aromatic acids. This acylation phenomenon typically occurs at the C_3_ position of the C ring, forming acylated anthocyanins. This multiplicity in structural modifications accounts for the existence of more than 500 identified anthocyanin-derived compounds, endowing anthocyanins with a broad spectrum of structural characteristics and physicochemical properties [10].

## 3. Physicochemical Properties of Anthocyanins

The physicochemical properties of anthocyanins are a function of their intricate molecular structure, featuring a dual benzene ring configuration and a densely packed molecular system. This molecular composition imparts notable polarity to anthocyanins, rendering them readily soluble in water and easily dissolvable in polar solvents such as methanol, ethanol, acetone, dilute alkali, and dilute acid, while they remain insoluble in nonpolar substances like ether, chloroform, and benzene. This solubility profile, coupled with their limited lipid solubility, contributes to their diminished ability to traverse phospholipid bilayers, thus resulting in reduced bioavailability [12,13].

When anthocyanins are extracted from botanical sources, they exhibit an exceptional susceptibility to degradation. This degradation is affected by various factors including acidity, temperature, their own concentration, exposure to light, oxygen, enzymatic activity, cofactors, microorganisms, and metal ions [14]. Consequently, the application of anthocyanins is somewhat restricted due to their inherent instability. As pigments, anthocyanins manifest distinct colors contingent upon pH levels. Specifically, they assume a red hue at pH values below 7, transition to purple in the pH range of 7 to 8, and appear blue when exposed to pH levels exceeding 11. This property underpins their ability to impart red, orange, purple, and blue hues to plant organs such as petals, fruits, and leaves. It is imperative to note that anthocyanins retain stability only in acidic conditions with a pH less than or equal to 3. At concentrations of hydrochloric acid equal to or greater than 0.12 mol·L^−1^, partial hydrolysis of anthocyanins occurs. Hence, during the extraction, isolation, or purification of anthocyanins, meticulous attention should be directed toward monitoring the concentration of hydrochloric acid. Moreover, alterations in pH can modulate the color intensity and chromaticity of anthocyanins without compromising the characteristic spectra of interfering substances [15]. Anthocyanins exhibit strong UV and visible region absorbance, with the maximum absorption wavelength occurring in the UV region and another peak in the visible region. This dual absorption feature, with its respective peaks at approximately 500–540 and 275 nm, is indicative of their spectral properties [16]. In their capacity as food colorants, anthocyanins possess a commendable safety profile, being non-toxic, non-allergenic, non-teratogenic, and non-carcinogenic.

**Figure 2:**
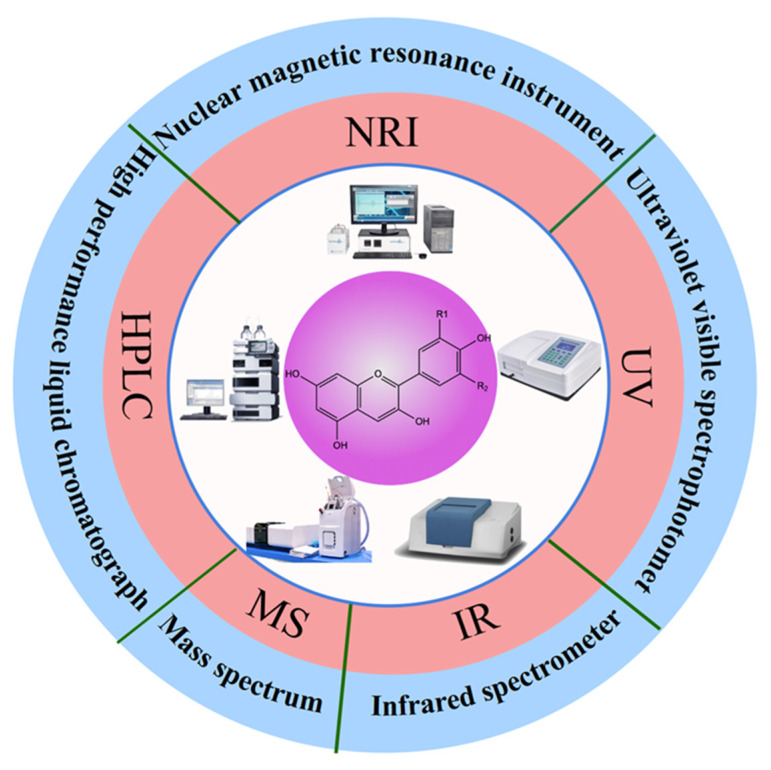
The qualitative identification and quantitative determination methods of anthocyanins.

The structural and compositional adaptability of anthocyanins allows for modifications, and enhanced comprehension of their structure, physicochemical attributes, and pharmacological effects empowers the development and production of analogous anthocyanin-like compounds. Significant alterations in the physicochemical properties of anthocyanins accompany structural or compositional changes. Various analytical techniques were developed for the quality and quantity of anthocyanins (Figure 2). UV-visible spectroscopy, a simple and cost-effective method, offers qualitative identification and quantitative determination of straightforward mixture systems [5]. Paper chromatography and thin-layer chromatography, employing basic equipment, enable swift qualitative identification of uncomplicated mixture systems. For more intricate analyses, high-performance liquid chromatography (HPLC) and UHPLC come to the fore [4]. These methods allow qualitative identification and precise quantification of anthocyanins with exceptional speed and sensitivity, albeit at a higher operational cost. In the case of highly polar, thermally unstable, and challenging-to-volatilize high-purity samples, mass spectrometry provides a powerful qualitative analysis tool, distinguished by its sensitivity and stability. Meanwhile, NMR spectroscopy, though characterized by a longer turnaround time and reduced sensitivity, facilitates the qualitative identification of unknown anthocyanins, isomeric compounds, and complex acylated anthocyanins within natural products. Infrared Spectroscopy, offering high specificity, enables the qualitative identification of anthocyanins containing benzene rings, oxygen-containing heterocycles, sugars, hydroxyl groups, and methoxy groups [17]. This method, marked by its expeditious nature, does come with a comparatively larger relative error. Given the multifaceted nature of anthocyanin analysis, employing a single technique proves insufficient for the precise description of their structure and composition. Consequently, a combination of multiple analytical methodologies is often necessitated to enhance accuracy.

## 4. Extraction and Production of Anthocyanins

The extraction and production of anthocyanins involve a range of methods, from traditional solvent extraction to advanced techniques like supercritical fluid extraction. Ultrasonic and microwave methods expedite the process, while pressurized solvent extraction improves efficiency. Enzyme extraction, fermentation, and genetic engineering offer innovative avenues for the production of anthocyanin. These extraction methods and production processes of anthocyanins are summarized in Table 1.

### 4.1. Solvent Extraction

The solvent extraction of anthocyanins leverages their solubility to effectively recover these compounds from plant sources, like purple potatoes, pomegranates, and grapes. Consequently, reagents featuring enhanced solubility for anthocyanins, such as methanol, ethanol, acetone, and other polar solvents, are conventionally employed in the extraction process. Furthermore, given the heightened stability of anthocyanins in acidic environments, acidic solutions are typically employed as extractants. Methanol and citric acid have been determined as the optimal extractant choices [18]. Nevertheless, the drawbacks of organic solvents, including heightened environmental impact and challenging recovery processes, have spurred the predominant utilization of water-based extraction methods in practical applications. In practice, a pragmatic approach often involves harnessing the respective advantages of both water and organic solvents through a combination of multiple methods for anthocyanin extraction. This multifaceted approach serves to enhance the efficiency of anthocyanin extraction and bolster the safety of the overall extraction process.

### 4.2. Ultrasonic Extraction

Ultrasonic extraction methodology capitalizes on ultrasonic waves to facilitate plant hydration, resulting in the expansion of cell wall pores rupture. This effect serves to enhance the interaction between the extractant and anthocyanins, thereby augmenting the extraction efficiency [6]. The merits of ultrasonic-assisted extraction encompass the reduction in extractant volume, shortened extraction durations, and diminished environmental pollutant output associated with the extractant. Nevertheless, it is noteworthy that at lower ultrasonic frequencies, there is a discernible elevation in anthocyanin extraction efficiency. Conversely, as ultrasonic frequency and duration increase, a portion of the anthocyanins undergo degradation. This degradation phenomenon primarily arises from the conversion of vibrational energy into heat during the extraction process. Given the heightened sensitivity of anthocyanins to elevated temperatures, this process can lead to the degradation of a portion of the anthocyanin [19].

### 4.3. Microwave Extraction

Microwave extraction is a method that uses microwave radiation to heat a sample solvent mixture to extract anthocyanins. This method uses microwave energy to rapidly, efficiently, and uniformly heat plant cells containing water, consequently inducing cell division [21]. This cell division leads to cell rupture, which allows for a more complete interaction between the cell contents and the extractant, potentially reducing both the extraction time and the volume of solvent required. Additionally, the elevation in temperature engenders an augmented diffusion effect, further enhancing the overall extraction efficiency. Pattrathip et al. [20] identified optimal parameters for microwave extraction, which included an ethanol concentration of 55%, a microwave power setting of 140 W, an extraction duration of 45 s, and the repetition of the extraction process eight times. It should be noted, however, that the application of microwave extraction is significantly limited by the inherent temperature sensitivity of anthocyanins. To reduce the risk of overheating and prevent the degradation of anthocyanins, medium power settings and longer extraction durations are often used.

### 4.4. Supercritical Fluid Extraction Method

Supercritical fluid extraction of anthocyanins is a technique that exploits the influence of pressure and temperature on the solubility of supercritical fluids for the selective extraction of specific constituents from solid or liquid matrices [22]. Prior to the extraction process, a pre-treatment of the sample with supercritical CO_2_ effectively eliminates the non-polar fraction of the solution, thereby reducing the presence of interfering substances. The union of CO_2_ and water culminates in the formation of carbonic acid, eliminating the need for additional acid addition to the solution and eliminating redundant steps for recovery and purification. The production process of this method meets strict safety standards and is free of toxic elements, making the resulting product suitable for use as a food additive. This process limits the exposure of anthocyanins to atmospheric conditions and light, thereby reducing their susceptibility to oxidative degradation. In addition, it is noteworthy that, despite the potential for higher temperature application in anthocyanin extraction, the prevailing majority of researchers have refrained from utilizing temperatures exceeding 40 °C in the course of their experiments involving this methodology [18]. Nevertheless, it is imperative to acknowledge certain limitations associated with this method, particularly its elevated production costs.

### 4.5. Pressurized Solvent Extraction

The process of pressurized solvent extraction, in which high pressure is employed to maintain the liquid phase at temperatures surpassing the boiling point, serves to disrupt hydrogen bonds between molecules while preserving the non-polar attributes of water molecules [26]. Simultaneously, this method enhances the solubility of compounds, diminishes solvent viscosity and surface tension, and heightens sample wetting and matrix penetration [23]. Given the application of the method at elevated temperatures, it is imperative to recognize the increased susceptibility of anthocyanins to temperature-induced degradation. Consequently, a delicate equilibrium must be upheld to optimize the anthocyanin yield while minimizing its thermal degradation. Despite the inherent limitations of this approach, its potential for streamlined and automated production holds immense appeal, particularly within the sphere of industrial manufacturing. Consequently, it has found widespread application in the extraction processes of numerous food products.

### 4.6. Enzyme Extraction

The enzyme-based extraction method relies on the use of microorganisms or their enzymatic by-products, taking advantage of the remarkable specificity inherent in enzymatic reactions. This specificity facilitates the targeted degradation and separation of specific constituents within the cell walls of anthocyanin-containing cells. This enzymatic action disrupts the structural integrity of plant cell walls, thereby fully exposing the bioactive components and, ultimately, increasing the efficiency and yield of anthocyanin extraction. Since cellulose is the major component of most plant cell walls and serves as the primary barrier to the dissolution of bioactive compounds within these walls, cellulase is the most commonly used enzyme to promote the dissolution of anthocyanins by disrupting the cellulose barrier, thereby improving the extraction rate of anthocyanins [24].

### 4.7. Fermentation

Currently, the predominant source of anthocyanins is extraction from natural plants, a method that is widely used but is susceptible to variability due to natural factors such as season, geographic region, and plant growth and development [27]. When large amounts of anthocyanins are required, synthetic methods need to be considered. Because the chemical synthesis principle of anthocyanin is not yet clear, the traditional chemical synthesis method is not suitable for the production of anthocyanin [28]. Currently, fermentation has emerged as a prominent method for anthocyanin synthesis. This approach utilizes cellular fermentation processes to generate cellular metabolites, which are subsequently extracted to obtain the desired product.

In terms of anthocyanin synthetic pathways, particular attention has been paid to the production of anthocyanins by plant tissues or cell cultures [29]. Plants inherently possess the genetic blueprint for anthocyanin synthesis and are adept at producing anthocyanins and a spectrum of anthocyanin derivatives. The use of careful screening and rejuvenation techniques for high-yielding cell lines can lead to significant increases, often multiples or even tens of times, in the production of the target product. For instance, Vincenzo et al. [30] reported that cell cultures of potatoes exhibited three to five times higher total anthocyanin levels than their tubers of the same variety. This approach also avoids the waste of non-essential plant parts and facilitates the conversion of plant-derived compounds into more potent pharmaceuticals. Nevertheless, it is essential to acknowledge the inherent challenges associated with tissue culture, including production instability, variable yields across generations, and product variability.

Furthermore, addressing the intricacies of natural product biosynthesis involves optimizing bioreactor variables such as light exposure, impeller design, and sample extraction methods [31]. Parameters like medium composition, airflow rate, agitation rate, light conditions, and temperature must be fine-tuned to enhance the biosynthetic yield of anthocyanins [32]. Controlling the conditions of plant tissue culture is an effective strategy to increase anthocyanin production. For instance, sugar content exhibited a positive correlation with anthocyanin production in cells. Elevated sugar-induced anthocyanin accumulation results from altered expression of regulatory and structural genes, particularly UDP-glucose and anthocyanin 3-O-glycosyltransferase, coupled with substantial reprogramming of signal transduction pathways [33]. Light conditions also exert profound effects on anthocyanin production, with blue light rapidly stimulating anthocyanin accumulation in fruits, while red light fosters proanthocyanidin synthesis by inducing LAR and ANR [34]. Temperature is also critical, requiring higher temperatures for cell propagation to accommodate cell development and growth, and lower temperatures for anthocyanin production to prevent tissue browning and protect anthocyanins from oxidation or thermal decomposition [35].

While the plant tissue culture method holds considerable promise, it is not without its challenges. For instance, the issue of yield instability across generations in tissue culture remains contentious and unresolved. The demanding culture conditions required for plant tissue culture impose a significant cost burden. Plant cells produce multiple anthocyanins, making their separation a formidable task using current extraction and purification techniques. Moreover, the presence of polyphenol oxidase (PPO) catalyzes oxidation, leading to an undesirable “browning effect” in plant anthocyanin extracts, culminating in the unwanted amalgamation of resulting compounds into brown pigments [31]. Xiran et al. [36] explored four major classes of inhibitors to mitigate enzymatic browning reactions, offering protective inhibition against these reactions.

### 4.8. Genetic Engineering

The rapid development of recombinant DNA technology, genome sequencing, DNA synthesis, metabolic network modeling, and the growing importance of enzymology have paved the way for metabolic engineering as a potent biotechnological tool for the production of industrially important compounds by microorganisms. The use of microbial cell factories for anthocyanin production has the potential to effectively overcome the current obstacles associated with the extraction and purification of natural anthocyanins. Anthocyanins are commonly synthesized by *Escherichia coli* [37], *Saccharomyces cerevisiae* [37], and *Corynebacterium glutamicum* [25].

In addition, flavonols are converted to dihydroflavonols, leucine anthocyanins, and, finally, to anthocyanins through the action of a series of enzymes, including flavonol 3-hydroxylase (F3H), dihydroflavonol 4-reductase (Dfr), and α-ketoglutarate and iron (II)-dependent anthocyanin synthesis enzymes (Ans) [38]. The production of anthocyanins in microbial systems is closely correlated with the construction of anthocyanin synthetic pathways and the enzymes that control anthocyanin metabolism. Due to the conserved nature of these gene-edited enzymes and their regulation across different organisms, the expression levels of enzymes from different plant sources differ [39]. Brady et al. [40] successfully co-expressed anthocyanin O-methyltransferase (AOMT) from different plant origins with Petunia anthocyanin synthase (PhANS) and Arabidopsis anthocyanin 3-O-glucosyltransferase (At3GT) in Escherichia coli, resulting in a substantial 21-fold increase in O-methylated anthocyanin titers using plasmid copies. Additionally, cofactors play a pivotal role in anthocyanin synthesis, primarily facilitating electron transfer and enzyme activation. Modulation of these cofactors is often achieved by overexpression of synthetic genes and suppression of catabolic genes [40].

Despite the significant progress, the production of anthocyanins by recombinant microorganisms grapples with several challenges arising from the absence of various native plant environments, such as plant cell inclusion bodies and intracellular pH fluctuations. Moreover, the intricate internal environment that governs plant growth and development poses an additional challenge. For example, while engineered bacteria offer rapid growth times, standardized genetic procedures, and easily scalable culture conditions, their anthocyanin production still carries substantial costs and yields lower titers compared to natural anthocyanins [41]. Furthermore, anthocyanins produced by engineered bacteria face stability issues due to the absence of pH regulation mechanisms inherent in plant cells and vesicles.

## 5. Stability of Anthocyanins

### 5.1. Light Stability

The conjugated double bond in anthocyanin gives the aromatic acyl group of the anthocyanin molecule the ability to absorb light energy and potentially release electrons. When the photon is absorbed by the anthocyanin molecule and breaks the conjugated double bond in the anthocyanin molecule, photodegradation occurs and the aromatic acyl group in acylated anthocyanins absorbs photons and reduces the sensitivity of the acylated anthocyanins to light [42]. Natural anthocyanins predominantly exist as colored flavin oxygen ions (AH^+^) in acidic media (pH < 2.5). However, as the H^+^ concentration of the solution decreases (pH > 3), the predominant form transitions to hemiacetal B, leading to a gradual shift in anthocyanin coloration toward colorlessness or pale yellow. Subsequently, the hemiacetal can undergo ring-opening and isomerization into cis-hydroxychalcone or (Z)-hydroxychalcone, both of which lack color. These compounds can further isomerize into (E)-chalcone through thermal or photochemical processes [43]. In the excited state, both uncomplexed and complexed anthocyanins undergo efficient and rapid deactivation via two primary pathways: excited state intermolecular proton transfer (ESPT) in free anthocyanins and charge transfer-mediated internal conversion in cochromatic anthocyanins [44]. Furthermore, exposure to light can deactivate these colorless hydration products upon UV excitation.

The pH of the solution containing anthocyanins significantly influences their photochemical degradation due to its effect on the form of the anthocyanins [45]. In acidic media, anthocyanins exhibit relatively higher stability under light, with chemically modified anthocyanins, such as acylated anthocyanins, demonstrating superior stability compared to their unacylated counterparts. Hu et al. [46] conducted experiments with four anthocyanins extracted from black wolfberry and Tangut white spurge. In a pH 3 solution, under light conditions at 35 °C for 10 days, unacylated anthocyanins from Lycium barbarum maintained 79% preservation in the dark and 64% preservation in the light, whereas acylated anthocyanins from Tangut white spurge retained nearly 90% of their content in the light.

However, the number of acyl groups in anthocyanins does not always show a direct positive correlation with their stability. Escher et al. [47] studied the effect of pH and light on blue pea anthocyanins, which contain significant amounts of polyacylated anthocyanins. They were stored in solutions at pH 3.6 and 5.4 in a photooxidation chamber at 32 ± 2 °C. Under light conditions, the anthocyanin retention rate was 34.4% and 48.3%, respectively, and anthocyanin retention in solutions kept in darkness exceeded 90%. Furthermore, the light stability of anthocyanins is influenced by additives in the solution. For instance, ascorbic acid has a scavenging effect on peroxide radicals formed by anthocyanins under light stress. Under the protective influence of ascorbic acid, the degradation rates of both acylated and non-acylated anthocyanins under light show different degrees of reduction [7]. Song et al. [48] reported that the addition of 100 ppm of ascorbic acid under light stress at 500 W/m^2^ min^−1^ reduced the degradation rates of purple sweet potato anthocyanin and grape anthocyanin from 2.739 × 10^−3^ to 1.589 × 10^−3^ min^−1^, highlighting the protective role of ascorbic acid against anthocyanin degradation.

In summary, chemical modifications are required to improve the light stability of anthocyanins. The pH of the solution, additives, and storage conditions must be taken into account to ensure the rational use of anthocyanins.

### 5.2. Thermal Stability

Anthocyanins, as food-based pigments, are inevitably subjected to various thermal processes during food processing, such as pasteurization, which serves to prevent spoilage and deterioration. Consequently, understanding the effect of temperature on anthocyanins is an important facet of understanding anthocyanin stability. In general, anthocyanins exhibit considerable instability, with thermal processes exceeding 50 °C resulting in partial or complete degradation. The thermal stability of anthocyanins is further influenced by the acidity level of the solution in which they reside, with relative thermal stability observed below pH 3 and increased instability at neutral pH levels [47,49].

Anthocyanins can be categorized into monomers, zwitterions, and polymers based on their degree of polymerization. Monomers represent the fundamental structural units, zwitterions consist of 2–10 monomers, and polymers comprise more than 10 monomers, all characterized by unsaturated double bonds. These structural features make anthocyanins susceptible to degradation at elevated temperatures, and their thermal stability is closely linked to specific chemical structures [50]. Two hypotheses have been proposed regarding the degradation mechanism of anthocyanins. Connor et al. [51] postulated that anthocyanins transform into base-like glucosides, which subsequently open to form chalcone glycosides. These compounds further evolve, giving rise to chalcone and its isomer α-diketone, eventually culminating in the formation of phenolic acids, aldehydes, cyano-3-glucoside, and geranyl-3-glucoside. This hypothesis suggests that the initiation of anthocyanin degradation involves the opening of heterocycles and chalcone formation, a process intensified by elevated temperatures. Another hypothesis suggests that the thermal degradation of anthocyanins begins with the hydrolysis of C_3_ glycosides, resulting in the synthesis of anthocyanins that mimic bases. Subsequent isomerization results in chalcone and its isomer α-diketone. The chalcone structure is then subject to further transformation, giving rise to coumarin glucoside derivatives. Eventually, this transformation causes the B-ring to be lost [52]. Furthermore, alterations in solution pH cause changes in the form of anthocyanins in solution and, subsequently, shift the thermal degradation pathway of these compounds. In summary, anthocyanins undergo degradation via two pathways, ultimately yielding aldehyde and benzoic acid derivatives or chalcone and coumarin, respectively.

Different heat treatment conditions applied to anthocyanins have varying degrees of influence on their degradation, significantly impacting the anthocyanin content of the final products. Mahsa et al. [53] observed a 20% reduction in anthocyanin absorbance when heated to 85 °C, and a 53% reduction at 95 °C. Burgos et al. [54] also found that total anthocyanin content decreased by 23% after heating for 20–25 min and by half after heating at 121 °C for 10 min. Moreover, the use of microwave treatment significantly reduced the anthocyanin content. In summary, various heating methods have a prominent impact on the half-life of anthocyanins.

Certain additives can improve the thermal stability of anthocyanins and protect their integrity at high temperatures. Factors such as metal ions, ascorbic acid, sugar, and proteins can impact the stability of anthocyanins. Kopjar et al. [55] investigated the prevention of thermal degradation of anthocyanins in blackberry juice through the addition of sugar, specifically sucrose, fructose, dextrose, and alginate, at varying concentrations. The juice samples were heated at 50 °C, 70 °C, and 90 °C for 1 and 2 h. The protective effect was assessed based on the degradation rate of anthocyanins. Alginate exhibited the greatest impact on anthocyanin stability, with samples containing added alginate exhibiting the lowest reaction rate and the longest half-life. Furthermore, proteins can enhance the stability of anthocyanins. Wu et al. [56] investigated the effect of yeast mannose protein on the thermal stability of anthocyanins at pH 7.0. The primary kinetics results indicate that there is degradation of anthocyanins at both 80 °C and 126 °C. The inclusion of mannose protein, however, extended the half-life of anthocyanins by 4–5 times after being heated for 30 min at 80 °C and 126 °C, while sustaining their antioxidant capacity.

**Figure 3:**
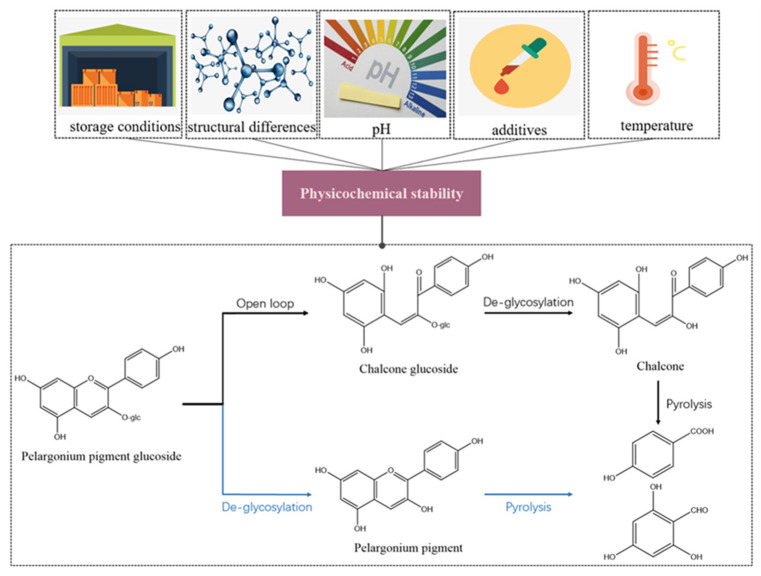
Schematic diagram of factors influencing physicochemical stability of anthocyanins and two special thermal degradation pathways of anthocyanins.

In brief, comprehension of how anthocyanins degrade at varying pH and temperature levels plays a critical role in minimizing such degradation during heat-based procedures (Figure 3). Additionally, incorporating the right additives can improve the thermal stability of anthocyanins, thus preserving their color and nutritional qualities.

## 6. Metabolism of Anthocyanins

### 6.1. Intake of Anthocyanins

Anthocyanins are present mostly in numerous fruits from certain plants, with predominant quantities in different grains and vegetables (such as eggplant, beans, cabbage, radish, and onions) along with an assortment of fruits such as strawberries, red cherries, plums, blackberries, raspberries, red currants, grapes, and blackcurrants. These compounds are primarily absorbed by the human body through the digestive tract [10]. Anthocyanins are commonly found in plant pulps but can also occur in the skin of fruits [57]. Their intake is closely linked to individual dietary habits, as the type and concentration of anthocyanins can vary among different foods.

Upon entering the oral cavity, anthocyanins initiate their journey through the digestive tract. Here, they undergo digestion and absorption into the bloodstream. The low bioavailability of anthocyanins, with plasma concentrations typically around 1% of consumption, may be attributed to the diverse range of digestive enzymes present in the digestive tract. Mallery et al. [58] discovered that consumption of black raspberry anthocyanins by healthy volunteers led to hydrolysis into their glycosidic form via B-glycosidases produced by both oral epithelial cells and bacteria. These conversion reactions, occurring during the brief period that food remains in the oral cavity, had a minimal impact on anthocyanin degradation.

The stomach is a crucial site for food digestion in the human gastrointestinal tract. It secretes digestive juices that contain acids and enzymes responsible for breaking down food [59]. Studies suggest that gastric acidity has little effect on anthocyanin degradation. Pepsin, an enzyme that turns protein molecules into peptides/amino acids, may have a limited effect on anthocyanin degradation, mainly by binding and reducing the anthocyanin content. The composition of gastric microbiota in healthy individuals is dynamic and influenced by various factors such as diet, medications, and disease. However, the effect of gastric microbiota on anthocyanin degradation and metabolism remains largely unexplored. It was found that anthocyanins can be absorbed through the stomach, and the absorption rate of anthocyanins is affected by factors such as culture time, pH conditions, anthocyanin structure, and molecular weight [60,61,62].

Any anthocyanins that are not metabolized and absorbed in the stomach move on to the intestine, which is the primary location for food digestion and nutrient absorption. The intestinal epithelium expresses several transporter proteins that facilitate the transfer of digested nutrients from the intestinal lumen to the bloodstream. These transporter proteins encompass hexose transporter proteins, ATP-binding transporter proteins (ABC), and monocarboxylate transporter proteins (MCT) [63]. Baron et al. [64] examined the pharmacokinetic profile of lingonberry anthocyanins in rats and their absorption via intestinal epithelial glucose transport proteins (sGLT1 and GLUT2). They found that after 30 min of ingestion, the peak blood concentration of anthocyanins in rats reached 11.1 ng/mL. Fasting conditions significantly increased anthocyanin bioavailability by more than sevenfold. Interestingly, various anthocyanins displayed distinct levels of bioavailability, whereby glycosides and glycosyl groups influenced absorption rates. Notably, there existed a significant correlation between the relative absorption of each anthocyanin and its molecular interactions with GLUT2 and SGLT1.

Today, individuals are aware of the benefits of fruits and vegetables in general, leading to systematic research on their ideal intake and associated health effects. Earling et al. [65] investigated the dosage of anthocyanins in acai fruit pulp, a rich source of anthocyanins, and 19 dietary supplements derived from it (including 6 capsules, 9 powders, and 4 concentrates) and found substantial variations in the total anthocyanin content among different products. For instance, capsule 6 (C_6_) contained a mean total anthocyanin content of 0.06 Ke mg/g, while capsule 1 (C_1_) had a content of 19.24 Ke mg/g. Statistically significant discrepancies in total anthocyanin distribution among various products were observed. Freeze-dried açai products such as capsules C_1_, and powders P_3_ and P_7_ exhibited the highest anthocyanin levels, while liquid supplements and frozen fruit pulp had the lowest concentrations. The quality of anthocyanin supplements available on the market can evidently vary significantly, as it is influenced by the employed production methods.

### 6.2. Metabolism of Anthocyanins

**Figure 4:**
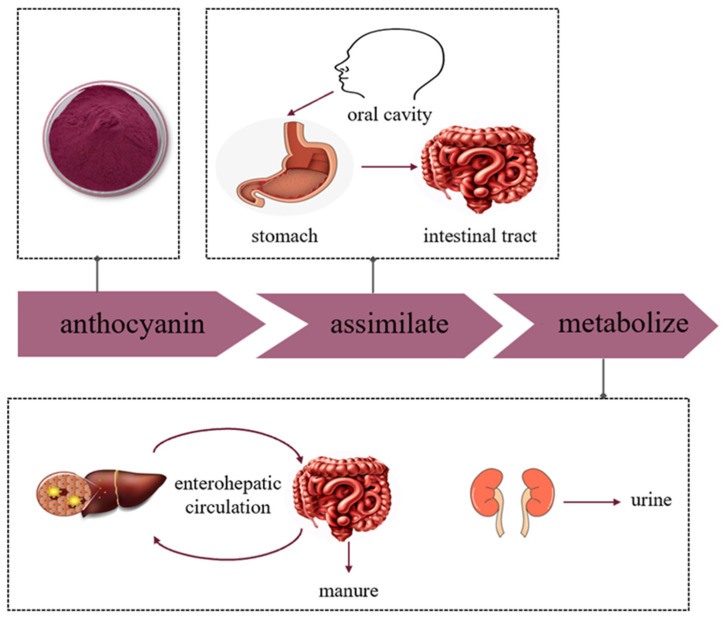
Schematic diagram of anthocyanin metabolism.

After being digested and entering the bloodstream, anthocyanins undergo substantial metabolism (Figure 4). Ferrars et al. [66] labeled anthocyanins and orally administered 6,8,10,3′,5′-13C5-C3G to participants. The researchers identified 34 metabolites, with 17 detected in the circulation, 31 in the urine, and 28 in the feces. These findings suggest that ingested anthocyanins undergo significant redistribution, transformation, or degradation within the body. Bile, which acts as a carrier for various substances, is produced in the liver and stored in the gallbladder. During food consumption, bile is released into the small intestine and can be reabsorbed via enterohepatic circulation. After absorption into the bloodstream, anthocyanins are conveyed through the portal vein to the liver and subsequently distributed to hepatocytes. After undergoing hepatic metabolism, anthocyanins re-enter the intestinal system via the bile. Once in the intestine, the intestinal microbiota transforms some anthocyanins into low-molecular catabolic metabolites, like phenolic acids and other phenolics. These metabolites may be reabsorbed or excreted through feces. After absorption, anthocyanins and their catabolic products undergo further metabolism. Specifically, these compounds are catalyzed by UDP-glucuronosyltransferase (UGTS), sulfate-transferase (SULTS), and catechol-O-methyltransferase (COMT) in the intestinal wall to form glucuronidation, sulfation, and methylation, respectively [67]. It should be noted that bile acids can circulate in the enterohepatic circulation multiple times, although the number of cycles for anthocyanins remains unknown [68]. Kalt et al. [69] examined anthocyanin metabolites in urine and identified over 200 such metabolites. These compounds, which are mainly glycosidic elements, show a wide range of polarity and are excreted in the urine, where they coexist with phospholipids rich in bile. Remarkably, high levels of anthocyanin metabolites persisted in urine even after five days without anthocyanin intake. Extensive enterohepatic circulation results in prolonged anthocyanin residence and considerable anthocyanin II binding. Anthocyanins are present in almost all tissues, including brain tissue where Phase II metabolites of anthocyanins have been identified [70]. Natural anthocyanins, however, are solely present in gastric tissues within the digestive system. Other organs, such as the jejunum, liver, and kidneys, contain both natural and modified anthocyanins, with varying proportions of anthocyanin derivatives in different organs. The liver exhibits the highest proportion of methylated forms, while glycosidic forms predominate in the jejunum and plasma [10].

The concentration and abundance of anthocyanins and their metabolites are critical factors influencing their potential physiological effects, as the bloodstream can transport anthocyanins to target sites where they may exert their effects [71]. The specific physiological effects of individual anthocyanin species vary based on their uptake, relative bioavailability, and distribution throughout the body.

For instance, anthocyanins distributed in the gut can interact with intestinal flora, altering the protective effects of anthocyanins against obesity and associated insulin resistance. Esposito et al. [72] investigated the gastrointestinal distribution of blackcurrant anthocyanins and their phenolic acid metabolites in obese mice consuming a diet supplemented with blackcurrant extract. It was observed that intact intestinal microbiota was essential for the reduction of weight gain and improvement of glucose metabolism in mice fed a low-fat or high-fat diet with blackcurrant extract. Administration of antibiotics substantially increased fecal anthocyanin content, indicating heightened sensitivity to intestinal microbial degradation. Moreover, gallic acid levels were found to be significantly higher than protocatechuic acid in the jejunum of both normal and antibiotic-treated animals, suggesting that this effect may be independent of intestinal microbiota status. Furthermore, these observations collectively emphasize the influence of gut microbiota and phloretin glycoside fraction in modifying the protective effects of anthocyanins against obesity and insulin resistance.

### 6.3. Bioavailability of Anthocyanins

The bioavailability of anthocyanins refers to the extent to which ingested anthocyanins are accessible to target tissues. This encompasses the release of anthocyanins from the food matrix and their absorption as they traverse the digestive tract [8]. The Food and Drug Administration (FDA) in the United States defines bioavailability as “the rate and extent to which the active ingredient or fraction is absorbed and becomes available at the site of action.” Methods for assessing the bioavailability of anthocyanins encompass in vivo and in vitro systems, as well as experiments designed to emulate human behaviors [71]. Anthocyanin bioavailability indicates the degree of absorbability within the body when the ingested substance is small and a transport system is available.

The bioavailability of anthocyanins appears to be lacking, with estimates ranging from 0.26% to 1.8% when given intravenously. This mode of administration only leads to a minor accumulation of anthocyanins in the target organ or circulatory system [73]. It is important to note that bioassays that solely target parental compounds and/or phenolic acid breakdown products may underestimate the actual values of bioavailability [10]. Upon evaluation of unaltered parental compounds, as well as phase I and II metabolites, conjugated products, and metabolites generated by microorganisms, it is apparent that the overall bioavailability greatly exceeds previous estimations. However, ongoing research is needed to determine the specific roles these metabolite categories play in maintaining human health [74].

The bioavailability of anthocyanins varies significantly among individuals, resulting in diverse results in human experiments on multiple occasions. Previous research reveals that anthocyanin bioavailability is predominantly associated with three key factors: the food matrix and food processing, anthocyanin metabolism and transport enzymes, and intestinal microorganisms [67].

The significant influence of the food matrix and food processing on anthocyanins is due to their susceptibility to structural alterations as discussed earlier. During food processing, temperature and pH are critical factors that can considerably diminish anthocyanin content in foods. Furthermore, changes in anthocyanin structure influence their bioavailability. Indeed, the structural composition of anthocyanins plays a pivotal role in determining their bioavailability. Notably, acylation markedly heightens the challenge of anthocyanin bioavailability. The content of anthocyanin is determined by the plant variety and the food matrix. Other food components can enhance or impede anthocyanin bioavailability. The handling of anthocyanin-rich foods, including factors such as pH, temperature, light conditions, and metal ions, affects both the content and structure of anthocyanin. Wiczkowski et al. [75] investigated the effect of the fermentation process on anthocyanin bioavailability in red kale. In a randomized crossover study, 13 participants consumed fresh and fermented red kale. Blood and urine samples collected before and after consumption revealed that fresh red kale anthocyanins exhibited over 10% higher bioavailability compared to their fermented counterparts. This study underscored the impact of food processing on anthocyanin bioavailability.

It is well-established that anthocyanins undergo transformation by various enzymes in the digestive system, significantly affecting their bioavailability in the body. For instance, glucuronide structure transferase (UGT) exhibits variability in glucuronide binding for anthocyanins among individuals, thereby influencing individual anthocyanin bioavailability. Court et al. [76] analyzed data from human liver banks to explore hepatic UGT. It was found that the ranking of activity variation was UGT1A1 > 1A6 > 2B15 > 1A4 = 1A9 > 2B7, with coefficients of variation ranging from 92% to 45%. The variation in UGT activity was attributed to donor age, sex, enzyme inducers, and genetic polymorphisms. Importantly, enzyme inducers such as co-administered drugs, smoking, and alcohol could increase UGT levels in the liver.

The role of gut microbes is integral to the study of anthocyanin bioavailability, as they represent one of the most influential factors in anthocyanin absorption. These microorganisms use anthocyanin metabolism to produce new anthocyanin metabolites that can be taken up by the intestinal epithelium, leading to physiological regulation. Therefore, the bioavailability of anthocyanins is largely dependent on the capabilities of the intestinal microbiota for biotransformation. Given the interindividual variability in intestinal microbiota, differences in anthocyanin responses are to be anticipated among individuals. Chen et al. [77] explored anthocyanin biotransformation properties using five intestinal probiotics. These probiotics, namely *Lactobacillus acidophilus* GIM 1.83, *Lactobacillus bulgaricus* GIM1.155, *Bifidobacterium animalis* GIM1.169, *Lactobacillus plantarum* GIM1.35, and *Streptococcus thermophiles* GIM1.321, were incubated with mulberry anthocyanins under anaerobic conditions at 37 °C. The study revealed that all strains exhibited some degree of anthocyanin conversion, with *Streptococcus thermophilus* GIM 1.321 and *Lactobacillus plantarum* GIM1.35, boasting stronger β-glucosidase production capacities, achieving degradation rates of 46.17% and 43.62%, respectively. Anaerobic processing primarily transformed mulberry anthocyanins into chlorogenic acid, cryptophyllic acid, caffeic acid, and ferulic acid. This experiment confirmed the degradation of various types of mulberry anthocyanins and identified the resulting metabolites from anthocyanin conversion. It highlights the significant impact of microorganisms on anthocyanin bioavailability.

## 7. Physiological Functions of Anthocyanins

### 7.1. Antioxidant Effect of Anthocyanins

Anthocyanins, as a class of biologically active compounds, exert significant influence on human health, primarily attributed to their potent antioxidant capabilities. The salient feature among their myriad effects is their capacity to neutralize reactive oxygen species (ROS) directly, while also eliciting indirect pathways to stimulate the synthesis or biological activity of crucial antioxidant enzymes, including catalase, superoxide dismutase (SOD), and glutathione peroxidase. Additionally, anthocyanins are capable of inhibiting enzymes responsible for the generation of ROS, such as nicotinamide adenine dinucleotide phosphate (NADPH) oxidase, while also regulating the slight uncoupling of mitochondrial respiration to prevent ROS production [2]. The therapeutic effectiveness of anthocyanins is heavily dependent on their ability to scavenge ROS and their regulatory role in the cellular antioxidant system.

Free radicals, including ROS and reactive nitrogen species (RNS), are essential to normal bodily function, with redox reactions maintaining their equilibrium. However, oxidative stress can occur when this balance is disrupted, leading to an imbalance that plays a vital role in the development and progression of chronic diseases such as cardiovascular disease, cancer, and aging [78]. Anthocyanins often act as donors of hydrogen ions or employ single-electron transfer mechanisms to thwart oxidation, thereby mitigating oxidative stress by scavenging free radicals and inhibiting their production [79]. As a result, the structural features of anthocyanins greatly affect their antioxidant capacity.

The antioxidant capacity of anthocyanins is tied to the arrangement of their rings, which determines proton transfer ease. The number and positioning of free hydroxyl groups on the pyranone ring also affect their antioxidant action [80]. Research revealed that flecainide exhibits the highest antioxidant activity among six anthocyanins due to the presence of three hydroxyl groups on its B ring [81]. Furthermore, other structural elements incorporated in the anthocyanin framework significantly enhance their antioxidant abilities, with glycosylated B-ring structures being particularly noteworthy. In addition, adjacent hydroxylation and methoxylation further intensify the antioxidant activity.

Physical factors have a significant impact on anthocyanins’ antioxidant properties. Different extraction techniques applied to the same source can produce anthocyanins with varying activities due to variations in operating procedures and conditions. It was found that solvent extraction achieved the highest average antioxidant inhibition rate at 90%, followed by enzyme extraction at 80%, and ultrasonic extraction at a mere 60%. This underscores the potency of solvent extraction in maximizing the antioxidant potential of blueberry anthocyanins [82]. Since anthocyanins rely on single electron transfer and hydrogen ion transfer mechanisms for their antioxidant effects, temperature, and pH are crucial factors that influence their effectiveness. Shi et al. [83] found that anthocyanins exhibited enhanced free radical scavenging ability as the temperature increased within the pH range of 1–8.

### 7.2. Immunomodulation by Anthocyanins

Two important aspects supporting the immunoregulatory function of anthocyanins revolve around their capacity to regulate oxidative stress and inflammatory responses. The sustained consumption of foods rich in anthocyanin yields a potent effect that does not solely rely on the chemical antioxidant properties of consumed anthocyanins; rather, this health-promoting process often corresponds to the organism’s adaptation to anthocyanins [84].

Inflammation is a crucial protective mechanism that the body uses to combat external agents like bacteria, aiding in the elimination of harmful stimuli and the initiation of the healing process. However, inflammatory responses can facilitate host ailments, including hay fever, glomerulonephritis, atherosclerosis, and gastroenteritis, and may also promote cancer development under conditions of aberrant immune function. Organisms meticulously regulate inflammation by overseeing the secretion of inflammatory cytokines and mediators. Disease progression often commences with the engagement of inflammatory factors (e.g., histamine, leukotrienes, PGE2, bradykinin), characterized by substantial vascular effects such as heightened permeability, vasodilation, sluggish blood flow, ultimately culminating in leukocyte aggregation and extravasation into the vasculature. This process is accompanied by the secretion of cytokines like TNF-α, IL-6, and IL-1β by granulocytes, along with the promotion of other inflammatory mediators and macrophage recruitment to the site of inflammation [85].

Anthocyanins, a class of compounds with immunomodulatory effects, have a significant role in regulating protein expression in the body through various cellular mechanisms that reduce oxidative damage and inhibit inflammatory responses in cells. These mechanisms encompass the upregulation of cytosolic cyclic adenosine monophosphate (cAMP), induction of antioxidant enzymes such as superoxide dismutase and catalase through nuclear oxidation reducers/antioxidant response elements (Nrf2/ARE), and the downregulation of nuclear factor kappa light chain enhancers in activated B cells [86]. The beneficial effects of consuming anthocyanin-rich foods as part of a healthy lifestyle seem to depend on the activation of specific cellular pathways that enable the development of a flexible cellular microenvironment with antioxidant and anti-inflammatory properties. This microenvironment can effectively respond to immune activities triggered by diverse physiological (e.g., exercise) or pathophysiological events [87]. André et al. [88] discovered that apple cultivars with a high concentration of proanthocyanidins significantly inhibited NF-κB, while the fraction with high levels of triterpenes considerably decreased the promoter activity of the TNF-α gene. This research offers fresh insights into leveraging apple genetic diversity to combat inflammation, particularly in the context of diseases associated with acute or chronic inflammation, such as neurodegenerative disorders linked to tumor necrosis factor [89].

Furthermore, anthocyanins not only reduce inflammatory responses but also modulate gut microbiota, indicating a broader impact on health. Espley et al. [90] conducted experiments involving male mice, comparing genetically modified apples with elevated flavonoid content to non-genetically modified apples with standard flavonoid levels. The research investigated the effects of dietary flavonoids on both inflammatory responses and gut microbiology in mice. The results indicated that mice on a flavonoid-rich diet exhibited a reduction exceeding twofold (*p* < 0.05) in transcript levels of inflammation-related genes, including interleukin-2 receptor (Il2rb), chemokine receptor 2 (Ccr2), chemokine ligand 10 (Cxcl10), and chemokine receptor 10 (Ccr10). Additionally, these mice showed a 6% increase in total bacterial count compared to the control group.

Studies have elucidated the association between the chemical structure of anthocyanins and the molecular pathways governing their anti-inflammatory responses. Specifically, their effectiveness as anti-inflammatory agents depends on their glycosyl and glycoside structures, as well as patterns of hydroxylation and methylation. Triebel et al. [91] probed the effects of lingonberry extract (BE) and anthocyanins on pro-inflammatory genes in human colonic epithelial cells stimulated with IFN-γ/IL-1β/TNF-α. It was found that BE and individual anthocyanins significantly suppressed the expression and secretion of pro-inflammatory mediators, such as TNF-α, IP-10, I-TAC, sICAM-1, and GRO-α, in stimulated cells. Interestingly, anthocyanins showed greater stability, which probably enhanced the effectiveness of BE.

### 7.3. Cancer Prevention

The prevailing theory of cancer suggests that it results from somatic cells multiplying uncontrollably due to the gradual accumulation of random mutations in crucial genes regulating cell differentiation and growth [92]. The onset of these mutations is initiated by the assault of free radicals on DNA. When DNA repair mechanisms falter, mutations gradually accumulate. Subsequently, when the oncogenes within these cells are silenced, cancer cells commence their development. Complementing this theory is the concept of “immunosurveillance,” which proposes that successful evasion of the immune system by accumulated mutated cells leads to uncontrolled tumor growth. One significant factor contributing to the increased incidence of cancer among immunosuppressed and immunodeficient individuals is the absence of immunosurveillance.

The antioxidant properties of anthocyanins can decrease the occurrence of mutations by reducing the generation of free radicals. Cristóbal et al. [93] reported that the effects of crude extracts of Carafate fruit and anthocyanin-rich extracts on human gastric cancer cell lines (agc) and gallbladder cancer cell lines (g415) were explored in vitro. These extracts exhibited substantial antioxidant activity, with Carafate berry extracts rich in anthocyanins demonstrating noteworthy potential. The anthocyanin-rich extracts demonstrated a higher effectiveness in inhibiting both the activity and migration of gastric cancer and gallbladder cancer cell lines when compared to the crude extract.

Marty et al. [94] proposed that genetic factors play a crucial role in regulating tumorigenesis by influencing the immune system’s ability to recognize specific common transformations. This suggests that individual differences in immune system expression may be associated with cancer susceptibility. In contrast, anthocyanins and their metabolites can provide protection against esophageal cancer through the consumption of anthocyanin-rich fruits. These compounds inhibit cell proliferation, dampen inflammation, induce apoptosis, promote cell differentiation, and enhance cell adhesion. Moreover, they restrict the production and progression of cancer by regulating several genes expressed through different signaling pathways, thus protecting cellular functions. Daniel et al. [95] investigated the role of protocatechuic acid (PCA), the principal microbial metabolite of black raspberry (BRB) anthocyanins, in preventing NMBA-induced esophageal cancer in rats. The results indicated that BRBs, their constituent anthocyanins, and PCA effectively inhibited esophageal tumor formation through their suppressive effects on inflammation-related genes. Elisabeth et al. [96] found that a diet enriched with 10% anthocyanins from lingonberry extract had anti-inflammatory and chemopreventive effects on cancer by reducing colon inflammation.

High-risk behaviors and environmental factors play a vital role in cancer development. Factors associated with the etiology of esophageal squamous cell carcinoma, including smoking, alcohol consumption, dietary habits, and human papillomavirus infection, can markedly increase the risk of cancer development. Conversely, a healthy diet has been found to considerably lower the incidence of esophageal cancer [97].

Moreover, anthocyanins can inhibit cancer progression by modulating the expression of specific genes involved in cancer cell spread. Li et al. [98] examined the anti-metastatic properties of anthocyanins from black rice on ErbB2-positive breast cancer cells. The findings revealed that these anthocyanins reduced tumor growth, inhibited lung metastasis, and decreased the activity of the metastasis-promoting factor urokinase-type fibrinolytic plasminogen activator (u-PA). The concentration-dependent effect of anthocyanins inhibited the migration, adhesion, motility, and invasive ability of breast cancer cells.

### 7.4. Anthocyanins Regulate the Cardiovascular System

Diseases that impact the cardiovascular system, such as coronary artery disease, hypertensive heart disease, heart failure, and cerebral thrombosis, are significant public health concerns. The development of cardiovascular system diseases is chiefly attributed to detrimental lifestyle habits, including but not limited to smoking, alcohol misuse, sedentary behavior, and unhealthy dietary patterns. Multiple pathological mechanisms contribute to the progression of cardiovascular system diseases, with risk factors such as hyperlipidemia, obesity, hypertension, atherosclerosis, oxidative stress, inflammatory responses, and hyperglycemia intricately linked to these maladies [99]. While pharmaceutical interventions have made progress in extending disease progression and improving patient quality of life, they often come with significant adverse effects, including gastrointestinal complications, myocardial degeneration, and arrhythmias [100,101]. Conversely, mounting evidence underscores the myriad biological activities inherent in natural foods rich in polyphenols, with a paramount focus on their antioxidative and anti-inflammatory properties as pivotal agents in the prevention of cardiovascular diseases. Epidemiological investigations probing the relationship between dietary habits and cardiovascular ailments have consistently demonstrated that increased consumption of fruits, vegetables, grains, and nuts correlates with a diminished risk of cardiovascular disease [102].

Anthocyanins, which are naturally occurring polyphenolic compounds present in a variety of foods, have a significant impact on lowering oxidative stress and alleviating inflammatory reactions. They also possess an exceptional ability to neutralize risk factors linked to cardiovascular disease, including the ones stemming from atmospheric pollutants. At the genetic level, anthocyanins can significantly ameliorate risk factors associated with cardiovascular diseases by regulating the expression of inflammatory genes and reducing blood glucose, triglycerides, cholesterol, and low-density lipoprotein levels. Anahita et al. [103] discovered a noteworthy decrease in fasting glucose, triglycerides, cholesterol, and LDL levels in the group who followed a diet enriched with berry-based anthocyanins, in comparison to the control group. Importantly, PPAR-γ gene expression was positively correlated with lipid and glucose metabolism while negatively correlated with FBG, cholesterol, TG, and LDL levels. Furthermore, anthocyanins reduced serum HS-CRP levels and downregulated NF-κB-dependent genes, including TNF-α, IL-6, IL-1a, PCAM-1, and cox-2, in both metastatic and control subjects. These findings underscore the potential of berry supplementation in improving metabolic syndrome and associated cardiovascular risk factors, largely attributed to the inhibition of NF-κB-dependent gene expression and enhancement of PPAR-γ.

A diet rich in fruits and vegetables holds promise for improving heart health by reducing oxidative stress and modulating signaling pathways. Xu et al. [104] examined the potential of anthocyanins to prevent accelerated aging of mouse hearts. The study involved administering D-galactose to mice for accelerated aging, followed by anthocyanin intervention at 20 and 40 mg/kg. The evaluation of heart function and structure after 8 weeks revealed that anthocyanins delayed the reduction of cardiac index and myocardial tissue damage. Furthermore, they maintained the stability of redox systems, including GSH-Px, T-SOD, and MDA, both in serum and myocardial tissue. In the DNA damage signaling pathway, anthocyanins downregulated the expression levels of receptors such as ATM and ATR, along with Chk1 and Chk2 effectors. These findings underscore the potential of anthocyanins as nutraceuticals to delay age-related deterioration of cardiac structure and function by inhibiting DNA damage.

Atmospheric pollutants are major risk factors for cardiovascular disease. Inhaling particulate pollutants, such as PM2.5, triggers pulmonary inflammatory and pro-oxidant pathways that result in a systemic inflammatory response. This compromises vascular function and accelerates atherosclerosis. Wang et al. [105] investigated the protective effects of blueberry anthocyanin extracts against PM2.5-induced cardiac injury. Rats exposed to PM2.5 and treated with three different doses (0.5, 1.0, and 2.0 g/kg) of blueberry anthocyanin extract exhibited improved cardiac function, reduced cytokine levels, and enhanced levels of interleukin-10, cardiac tissue superoxide dismutase activity, and Bcl-2 protein expression. Additionally, levels of interleukin-6, malondialdehyde, endothelin-1, and angiotensin II were decreased, while Bax protein expression was reduced. This study proposes that a particular amount of blueberry anthocyanin extract has the potential to protect the cardiovascular system against the harmful effects of PM2.5.

Moreover, the salutary effects of anthocyanins extend beyond protection against injury. In healthy individuals, anthocyanins exert a protective influence on blood vessels, thereby enhancing vascular health and preserving dynamic homeostasis. Brett et al. [106] posited that blueberry anthocyanins possess substantial evidence of vascular protective effects. This evidence encompasses epidemiological, animal experimental, and human studies, all converging to demonstrate that blueberry anthocyanins enhance vascular health by targeting various aspects of the cardiovascular system. This includes reducing oxidative stress, modulating signaling pathways, and ameliorating dyslipidemia, resulting in potential physiological benefits for cardiovascular health.

## 8. Encapsulation of Anthocyanins

Anthocyanins are highly sensitive to environmental factors, such as light, heat, and oxygen, which can lead to their degradation and loss of bioactivity [107]. To preserve and effectively deliver anthocyanins for various applications, numerous encapsulation techniques and delivery systems have been explored. This section delves into the diverse strategies for anthocyanin encapsulation, several delivery vehicles are summarized in Table 2.

Emulsions are colloidal systems consisting of two immiscible phases, typically oil and water, stabilized by an emulsifying agent [116]. They have been widely employed to encapsulate anthocyanins, protecting them from degradation. Recently, microemulsion and nanoemulsion gained more and more attention. They are usually produced under high-speed shear, high-pressure homogenization, dynamic high-pressure microjets, and ultrasonication, and are often stabilized by surfactants and cosurfactants [109]. They have smaller particle sizes and are more stable, as well as the higher encapsulation efficiency of anthocyanins. Rabelo et al. [110] successfully formulated a food-grade water-in-oil (W/O) nanoemulsion to encapsulate anthocyanins, and it can remain relatively stable, with no phase separation after 30 days of storage; the retention rate (94.6%) of anthocyanins in nanoemulsions was higher than that (47.9%) of free anthocyanins. Nazareth et al. [109] found that the degradation kinetics of anthocyanins in microemulsion/nanoemulsion followed first-order reaction rates, and the half-life of anthocyanin in nanoemulsion (40 days) was longer than that of microemulsion (25 days) and free anthocyanin (6 days). It indicated that nanoemulsion might have better storage stability and protection effects than microemulsion. In general, the hydrophilic anthocyanins are accommodated in the aqueous phase. To gain the higher encapsulation efficiency and better protection effect stable of anthocyanins, the water-in-oil-in-water (W_1_/O/W_2_) double emulsions were developed, which were made up of water-in-oil (W_1_/O) emulsions distributed in the outer aqueous phase (W_2_) as droplets [111]. Ozcan et al. [112] prepared a W/O/W emulsion with a high encapsulation efficiency of anthocyanins (87.89%) and found it also can improve the bioaccessibility of anthocyanins. Some studies have reported that anthocyanins in the emulsions had a protective effect against lipid oxidation [108], and the anthocyanin-loaded W/O/W emulsions exhibited good performance as a potential ink for food 3D printing. Recently, a new type of emulsion has been studied, namely Pickering emulsion, it avoids the use of surfactants, inhibits droplet coalescence, and possesses super stability [113]. Ju et al. [113] used the soy protein–anthocyanin complex nanoparticles as an interfacial stabilizer to produce a Pickering emulsion, and indicated that the Pickering emulsion exhibits unique characteristics, including extraordinary emulsion stability, improved oxidative stability, and resistance to in vitro digestion. Emulsions containing anthocyanins have shown good promise in various food and pharmaceutical applications.

Nanoparticles, including polymeric nanoparticles and lipid nanoparticles, have gained attention as efficient carriers for anthocyanin delivery. Polymeric nanoparticles, often made from biocompatible materials like β-Lactoglobulin [114], alginate, and chitosan, provide a protective matrix for anthocyanin encapsulation. A previous study by He et al. [14] found that the loading of anthocyanins on chitosan nanoparticles can prevent anthocyanin degradation in gastrointestinal fluids and improve its stability in a beverage. The stability of the encapsulated anthocyanins was 84.5% after 35 days at 4 °C, which was higher than that (71.2%) of unencapsulated anthocyanins [14]. Ge et al. [114] have found the composite nanoparticle composed of β-Lactoglobulin and chitosan derivatives was a good carrier for anthocyanins, it significantly improved the stability and bioavailability of anthocyanins in the simulated gastrointestinal tract. Lipid nanoparticles are composed of solid lipids and are usually used to encapsulate hydrophobic bioactive compounds. Some core–shell lipid nanoparticles were prepared by the dilution of water in oil (W/O) microemulsions containing a hydrophilic core in aqueous media. They can offer a stable environment for hydrophilic bioactive compounds, such as anthocyanins. Ravanfar et al. [115] prepared the solid lipid nanoparticles based on palmitic acid, span 85, and egg lecithin. These lipid nanoparticles have a high entrapment efficiency (89.2 ± 0.3%) and gain the advantage of controlled release and sustained delivery of anthocyanins.

In conclusion, anthocyanin encapsulation is a dynamic field that continues to evolve with the development of innovative delivery systems. These systems, including emulsions, microemulsions, nanoemulsions, biopolymer nanoparticles, solid lipid nanoparticles, and other vehicles, play a pivotal role in protecting anthocyanins from environmental stressors and enhancing their bioavailability. The choice of delivery system depends on the specific application and the physicochemical properties of the anthocyanin of interest. Further research in this area holds the potential to unlock new avenues for utilizing anthocyanins in various industries, ranging from food and nutrition to pharmaceuticals and cosmetics.

## 9. Conclusions

Anthocyanins are a class of bioactive compounds abundant in nature that offer significant nutritional value due to their widespread availability, affordability, and high public acceptance. They exhibit diverse biological activities, including potent antioxidant properties, immunomodulatory effects, regulation of blood lipid profiles, and a critical role in cancer prevention. While extraction and production methods for anthocyanins have advanced, their poor physicochemical stability remains a challenge, requiring a strategic focus on preserving their biological potency. Intensive research into process-resistant anthocyanin-based formulations in food science is needed, such as nanotechnology, multi-component synergistic effect, and colloidal delivery systems to improve their resilience and maximize their utility in food applications, with innovative approaches to maintaining anthocyanin functionality having the potential to stimulate market growth. The advancement of anthocyanin research through scientific investigation and technological intervention promises to unlock new opportunities for their application to improve human health and well-being.

## Figures and Tables

**Table 1:** The extraction and production method of anthocyanins.

Method	Raw Material	Advantages and Disadvantages	References
Solvent extraction	*Senggani* fruit	minimize damage or loss of compounds; low yield, low product quality, high energy input, time-consuming, need substantial amounts of solvents	[18]
Ultrasonic extraction	rose flower petal	significantly improve anthocyanins extraction rate, efficient; require complex and expensive equipment, difficult to realize industrialization	[19,20]
Microwave extraction	the fruits of *Syzygium nervosum* (ma-kiang in Thai)	green, rapid extraction time, higher extraction rate, with less organic solvent, wastewater removal, and energy use	[20,21]
Supercritical fluid extraction method	fruit wastes	green, reduced cost of operation; high cost, have limits to realize industrialization	[22]
Pressurized solvent extraction	black mulberry (*Morus nigra* L.) pulp	require less methanol consumption, higher extraction efficiency	[23]
Enzyme extraction	grape skins	enhance the yield, effective and environmentally friendly	[24]
Genetic engineering	*Corynebacterium glutamicum*	a sustainable method to produce anthocyanins, ease of cultivation and fast growth, availability of sophisticated genetic tools, and well-defined metabolic networks and models; the heterologous expression of plant-derived genes, optimal expression depends on the host strains	[25]

**Table 2:** Several delivery systems for the encapsulation of anthocyanin.

Type	Composition	Property	Function	References
Nanoparticle	β-cyclodextrin, chitosan hydrochloride, and carboxymethyl chitosan	particle size, 333.86 nm; zeta-potential, +45.97 mV	Improve the stability of anthocyanins, light irradiation for 12 days, the retention rate of encapsulated anthocyanin and free anthocyanin was 77.6% and 58.1%	[107]
Emulsion	bilberry seed oil	particle size, 1.7 nm; zeta-potential, −71.45 mV; isoelectric point, pH = 4.0	Display a protective effect against lipid oxidation	[108]
Nanoemulsion	organic phase (ethanol), aqueous phase (glycerol-sodium benzoate buffer solution)	particle size, 20–500 nm	Enhance the bioavailability and absorption, kinetically stable systems; thermal processing at 90 °C for 3 min, the retention of anthocyanins was 72.24%	[109]
Microemulsion	organic phase (ethanol), aqueous phase (glycerol-sodium benzoate buffer solution), and surfactant	particle size, 10–100 nm	Enhance the bioavailability and absorption, thermodynamically stable isotropic liquids; thermal processing at 90 °C for 3 min, the retention of anthocyanins was around 54%	[109]
Water-in-oil (W/O) nanoemulsion	a continuous oil phase, the dispersed phase, and an emulsifier	particle size, 131.5 nm to 195.3 nm; polydispersity (after 30 days of storage), 0.2 to 0.6	No phase separation after 30 days of storage, the samples had decreased concentrations of polyphenols after 30 days of storage at 4 °C, the highest retention was 94.6%	[110]
Water-in-oil-in-water (W_1_/O/W_2_) double emulsion	oil phase, the internal aqueous phase (W_1_), and the aqueous phase (W_2_)	particle size, 625 nm; zeta-potential, −48 mV; viscosity, 29.1 Pa·s	Improve the stability of anthocyanins, 28 days of storage at 4 °C and 25 °C, the retention rate of encapsulated anthocyanin were 93.2% and 88.9%	[111,112]
Pickering emulsion	soy protein isolate, anthocyanins	particles size, 186 nm to 675 nm	Notable improve radical scavenging activity and improved antioxidant capacity, the FFA releasing rate could be retarded by up to 6.9%	[113]
Nanocomplexes	chitosan hydrochloride, carboxymethyl chitosan, and β-Lactoglobulin	particles size, 91.71 nm; encapsulation efficiency, 69.33%	Improve the stability and bioavailability, anthocyanins retention rate was 68.9%	[114]
Chitosan nanoparticle	carboxymethyl chitosan, chitosan hydrochloride	particles size, 219.53 nm; encapsulation efficiency, 63.15%	Improve the stability of anthocyanins, after 35 days at 4 °C, the retention rate of encapsulated anthocyanin and free anthocyanin were 84.5% and 71.2%	[14]
Solid lipid nanoparticle	the lipid phase (palmitic acid, Span 85, and egg lecithin), cosurfactant (ethanol or isobutanol), and distilled water	particle size, 455 ± 2 nm; entrapment efficiency 89.2 ± 0.3%	Improve the stability of anthocyanins and enhances the oral bioavailability of anthocyanins	[115]

## Data Availability

The datasets generated for this study are available on request to the corresponding author.
